# Machine learning classification of autism spectrum disorder based on reciprocity in naturalistic social interactions

**DOI:** 10.1038/s41398-024-02802-5

**Published:** 2024-02-03

**Authors:** Jana Christina Koehler, Mark Sen Dong, Afton M. Bierlich, Stefanie Fischer, Johanna Späth, Irene Sophia Plank, Nikolaos Koutsouleris, Christine M. Falter-Wagner

**Affiliations:** 1 Department of Psychiatry and Psychotherapy, Medical Faculty, LMU, Munich, Germany; 2 Goethe University Frankfurt, University Hospital, Department of Psychiatry, Psychosomatic Medicine and Psychotherapy, Frankfurt am Main, Germany; 3 Max Planck Institute of Psychiatry, Munich, Germany; 4 Institute of Psychiatry, Psychology and Neuroscience, King’s College, London, UK

**Keywords:** Diagnostic markers, Autism spectrum disorders, Psychiatric disorders

## Abstract

Autism spectrum disorder is characterized by impaired social communication and interaction. As a neurodevelopmental disorder typically diagnosed during childhood, diagnosis in adulthood is preceded by a resource-heavy clinical assessment period. The ongoing developments in digital phenotyping give rise to novel opportunities within the screening and diagnostic process. Our aim was to quantify multiple non-verbal social interaction characteristics in autism and build diagnostic classification models independent of clinical ratings. We analyzed videos of naturalistic social interactions in a sample including 28 autistic and 60 non-autistic adults paired in dyads and engaging in two conversational tasks. We used existing open-source computer vision algorithms for objective annotation to extract information based on the synchrony of movement and facial expression. These were subsequently used as features in a support vector machine learning model to predict whether an individual was part of an autistic or non-autistic interaction dyad. The two prediction models based on reciprocal adaptation in facial movements, as well as individual amounts of head and body motion and facial expressiveness showed the highest precision (balanced accuracies: 79.5% and 68.8%, respectively), followed by models based on reciprocal coordination of head (balanced accuracy: 62.1%) and body (balanced accuracy: 56.7%) motion, as well as intrapersonal coordination processes (balanced accuracy: 44.2%). Combinations of these models did not increase overall predictive performance. Our work highlights the distinctive nature of non-verbal behavior in autism and its utility for digital phenotyping-based classification. Future research needs to both explore the performance of different prediction algorithms to reveal underlying mechanisms and interactions, as well as investigate the prospective generalizability and robustness of these algorithms in routine clinical care.

## Introduction

### Background

The diagnosis of autism spectrum disorder (ASD) encompasses a range of symptoms in reciprocal social interaction and communication as well as restricted, repetitive behaviors and interests [1]. The currently rising prevalence [2] exacerbates waiting times for an already long and demanding diagnostic process, increasing psychological stress on seeking diagnostic clarification [3]. Gold-standard recommendations include assessment with semi-structured diagnostic interviews or observational tools conducted by multidisciplinary teams, along with neuropsychological assessments and an anamnesis of developmental history by a caregiver [3]. With the increasing number of patients seeking diagnosis in adulthood, the lack of recommended diagnostic instruments for this population [4] poses an additional challenge. Therefore, the improvement of the diagnostic process of autism in adulthood has been named one of the top priorities in autism research [5].

Digitalized methods have high potential to improve screening and diagnostic procedures, such as assessing home videos [6] or interactions with virtual characters [7, 8]. While promising, these findings often rely on time-consuming manual behavioral coding or, more importantly, may not adequately reflect real-time social interactions, which are especially relevant for judging symptom strength [9]. Additionally, the increased use of artificial intelligence methods, such as machine learning (ML), has furthered research on increasing the efficiency of existing diagnostic tools, e.g., by identifying subsets of the most important items for diagnosis [10, 11], pointing to areas of impairments most indicative for diagnosis. These include aberrances in, e.g., gesturing, facial expressions and reciprocal social communication [10], traits which seemingly influence first impressions of people with ASD, who are judged as interacting more awkwardly by typically developing (TD) peers [12]. This suggests that non-verbal behavior also represents an important pillar of clinical impression formation.

Several computer vision approaches have been investigated to capture this different non-verbal behavior and explored its use for autism diagnosis, underlining its usefulness for the quantification of behavioral markers [13]. For example, using motion tracking, the degree of imitation of isolated hand movements could identify autistic and non-autistic adults with an accuracy of 73% [14]. A recent deep learning approach analyzing videos that depict clinical interviews of autistic and non-autistic children revealed a classification accuracy of 80.9% based on pose estimation [15].

### Reciprocal interaction in ASD

A way to quantify this aberrant interaction style is through closely examining the way two interacting partners temporally adjust their behavior with each other, or, in other words, how well they are “in sync”. Interpersonal synchrony or coordination can not only be achieved through mutual, bilateral matching, but also by establishing leader-follower relationships through unilaterally adapting to the behavior of the interactant [16]. Interpersonal synchrony has repeatedly been associated with rapport, affiliation, and perception [17, 18], emphasizing its importance for social cognition. In ASD, reduced interpersonal synchrony or coordination has in fact been described on multiple modalities and across the lifespan [19]. For instance, reduced coordination of emotional facial expressions has been found in autistic youth in conversation with a partner [20]. Interpersonal synchrony in head motion has been found to be reduced in diagnostic interviews with patients subsequently diagnosed with autism as compared to those who were not [21]. Another study investigating head and body motion synchrony in autistic and non-autistic adults found both to be reduced when an autistic person was part of the conversation [22], once again reflecting the importance of the interactional perspective. Further, synchrony and coordination differences in autism have also been found within the individual (intrapersonally), with reduced or differing coordination of simultaneous movements [23, 24] or across communication modalities [25]. Lastly, movement atypicalities, apart from coordination, appear to be pronounced in autism, including reduced facial expressiveness in autistic children [26], as well as a unique kinematic profile of biological motion and motor control [27]. A recent meta-analysis found a significant correlation between gross motor and social skills in autism [28], underlining the significance of movement differences for the core symptomatic profile of ASD.

### Aims

In summary, the mere definition of ASD as a disorder of social interaction implies an interdependency and calls for shifting to the dyad as unit of analysis [29]. However, feasible measures are lacking due to their reliance on extensive manual coding, experimental paradigms appearing staged or unnaturalistic, or investigating only isolated aspects of social interaction. Hence, the aim of this proof-of-concept study was to build upon existing knowledge of adaptation difficulties in autism and use the richness of non-verbal social interaction data in an efficient way to build an objective (i.e. independent of self- or clinician-ratings) classification model of autistic social interaction. To this end, we trained several Support Vector Machine (SVM) classification models to optimally differentiate between members of autistic vs. non-autistic interactional dyads. To increase objectivity and feasibility for potential further development in clinical practice, we used existing open-source algorithms that maximized automation in the annotation and analysis process.

## Methods

### Sample

We recruited 35 participants with ASD from a clinical database, as well as local autism networks. The diagnosis (F84.0 or F84.5) had to have been given by a qualified clinical psychologist or psychiatrist according to ICD-10 criteria as confirmed by a full diagnostic report. Inclusion criteria were an age between 18-60 years, normal intelligence (IQ > 70, as measured by an IQ score based on a verbal and non-verbal IQ test [30]) and no current neurological disorder. Additionally, 69 typically developing (TD) participants with no current or history of psychiatric or neurological disorders or psychotropic medication were recruited. Two ASD participants had to be excluded from the final sample because their diagnosis could not be verified on the basis of an incomplete diagnostic report. An additional five ASD participants were excluded during the analysis due to data loss from imprecise facial tracking. Due to the dyadic nature of the study, their interactional partners had to be excluded as well. Another TD-TD dyad was excluded due to technical issues during script loading, leading to a final sample of 88 participants. Groups were matched with respect to age and IQ. A chi-square-test of independence revealed no significant association between group membership and sex, *χ*^2^(1, *N* = 88) = 2.6, *p* = 0.11. A description of the final sample can be found in Table 1. All participants gave written informed consent before study participation and were compensated monetarily afterwards. The authors assert that all procedures contributing to this work comply with the ethical standards of the relevant national and institutional committees on human experimentation and with the Helsinki Declaration of 1975, as revised in 2008. All procedures involving human subjects/patients were approved by the ethics committee of the medical faculty of the LMU Munich (number 19-702).

**Table 1:** Sample description.

	ASD (*n* = 28, 18 female)	TD (*n* = 60, 26 female)	*p*_adjusted_
Age	37.18 (13.14)	31.48 (10.78)	0.101
Crystalline IQ	113.68 (16.90)	113.98 (16.69)	0.879
Non-verbal IQ	119.75 (23.44)	117.03 (17.37)	0.382

Mean parameter values (SD in parentheses) for each of the IQ tests for the ASD and TD participants, as well as the results of Wilcoxon tests (assuming unequal variances). Verbal IQ as measured by the Mehrfach-Wortschatz-Test. Non-verbal IQ as measured by the Culture-Fair-Test 20-Revised. Participants with ASD either had a diagnosis of F84.0 [3] or F84.5 [25]. *p* values adjusted for multiple comparisons using the false discovery rate (FDR) [69].

### Study setup

**Fig. 1:**
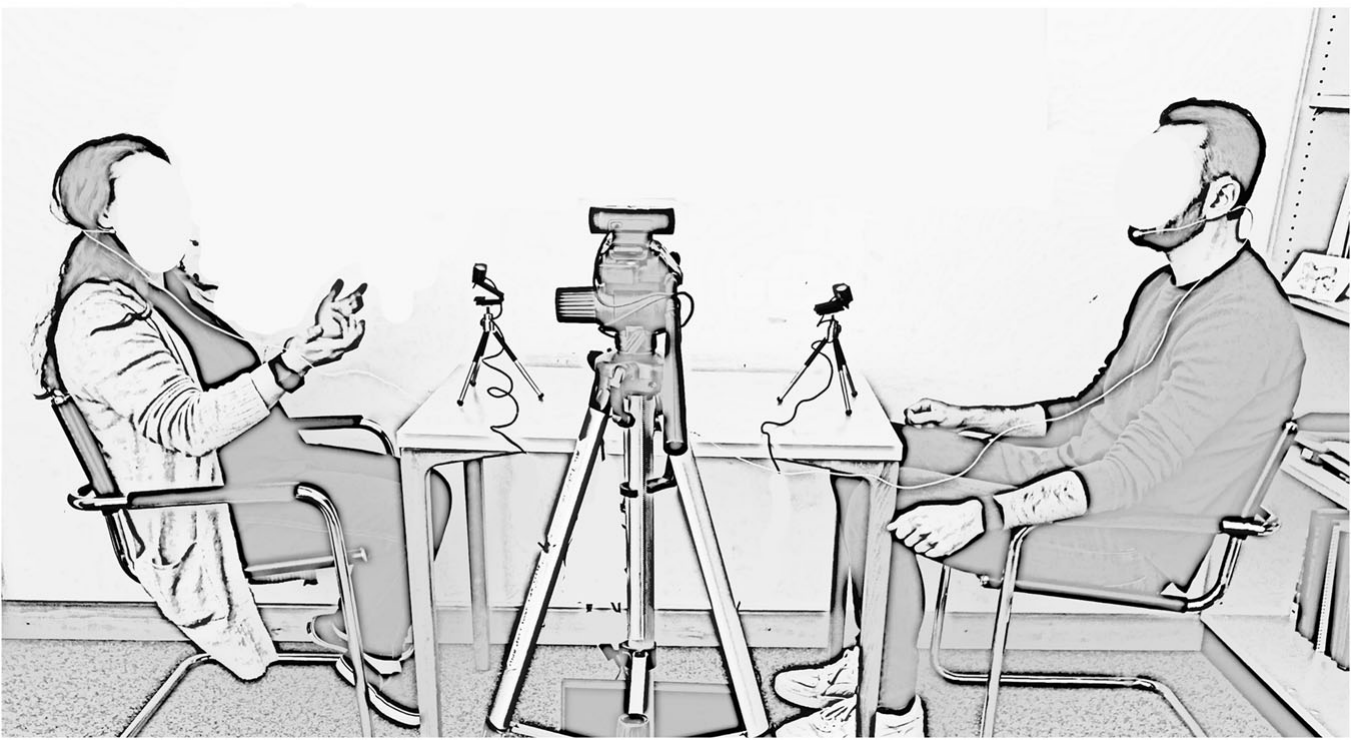
Experimental setup. Participants were seated across from each other and asked to conduct two conversational tasks. For additional setup info see Supplementary Material.

Participants were randomly paired resulting in 28 ASD-TD (mixed) and 16 TD-TD (non-autistic control) dyads. All were naïve to the diagnosis of their interactional partner. They were seated approximately 190 cm across from each other in fixed chairs. Two cameras (Logitech C922) were installed on a tripod on a table in front of the participants, recording their respective facial expression at 30 frames per second. A third camera was mounted at a wide angle on a tripod at a distance of approximately 240 cm (Fig. 1). All recordings were operated from a single computer using custom PsychoPy [31] scripts, allowing for maximal synchronization of the three video input streams. To control for any biases in subsequent video analyses caused by lighting change [32], measurements were taken in stable artificial light. To maximize hygienic safety measures during the Covid-19 pandemic, slight changes to the setup were required after the first nine participants were assessed (see Supplementary Information S4.3).

Participants engaged in two ten-minute conversation tasks for which they were instructed beforehand by the study personnel. After giving a starting cue with a clapping board, all study personnel left the room. Participants were asked to engage in a conversation about their hobbies, as well as to plan a fictional five course meal with dishes they both disliked. The mealplanning task has been used in previous synchrony studies (e.g., [22, 33]), with the rationale that a collaborative and funny task increases affiliation and synchrony respectively. As the overlap between dishes two people dislike tends to be smaller than finding common food preferences, this task requires more collaboration from both interactants. In contrast, a conversation about their hobbies was introduced. Restricted interests are a key diagnostic criterion of autism according to DSM-5 [1], whereby autistic individuals tend to switch to monologue style when talking about their interests [34]—a unique behavior, which we aimed to capture. The order of the tasks was counterbalanced among participants.

Additionally, participants completed a series of questionnaires to assess their level of self-reported autistic traits (Autism Quotient; AQ [35]), empathy (Saarbrücker Persönlichkeitsfragebogen; SPF [36], the German version of the Interpersonal Reactivity Index; IRI [37]), alexithymia (Toronto Alexithymia Scale; TAS20 [38]), depressiveness (Beck Depression Inventory; BDI [39]), self-monitoring (self-monitoring scale; SMS [40]), and movement difficulties (a German translation of the Adult Dyspraxia Checklist; ADC [41]). To obtain a best estimate of both their crystalline (Mehrfachwahl-Wortschatz-Intelligenztest; MWT [42]) and non-verbal (Culture-Fair Test; CFT 20-R [43]) IQ, two IQ assessments were undertaken, and their results averaged. Since difficulties in recognizing emotional facial expressions could potentially cause a bias in the investigation of synchrony in facial expressions, participants additionally completed a computer task for facial expression recognition (Berlin Emotion Recognition Test; BERT [44]).

### Data preparation and feature extraction

Videos were cut to a duration of ten minutes in DaVinci Resolve (Version 16.2.0054). Facial expression was analyzed with the open-source algorithm *Openface 2.0* [45], identifying action units (AUs) and three head pose parameters (pitch, yaw, roll) and extracting a time series of their presence and intensity for every frame. Motion Energy analysis (MEA [32]) was used to analyze head and upper body movement captured with the scenic camera. MEA extracts time series of grayscale pixel changes for every frame in pre-specified regions of interest (ROI). Due to the constant lighting conditions and a stable camera, pixel changes within each ROI indicate movement.

Prior to the final analyses, the behavioral time series from both tasks were synchronized between, and (in case of intrapersonal coordination) within, participants in the respective modalities. For this purpose, windowed cross-lagged correlations were computed in R. The size of the respective windows and lags for each modality were carefully chosen, relying on previous research wherever applicable [20, 46], to ensure maximum standardization. For the estimation of intrapersonal coordination, head movement, as derived from OpenFace, was cross-correlated with the body motion energy times series derived from MEA. Finally, summary scores (mean, median, standard deviation, minimum, maximum, skewness, and kurtosis) of the maximum synchrony instances from both tasks for each person were extracted. The extent as to which each person was synchronizing within the dyadic interaction was defined as their degree of imitating (following) their partners movements. For further details on the cross-correlation and feature extraction procedures, refer to Supplementary Information S2.

Facial emotion recognition capabilities were operationalized as mean accuracy (in %) and response time (in ms) (see Supplementary Information S3.4).

A full list of features can be found in Supplementary Table S13.

### Classification models

Separate SVM classification models were trained using features grouped according to the interaction modalities. The feature vectors for each participant combined the values from both the mealplanning and hobbies task. In each base model, the SVM algorithm independently modeled linear relationships between features and classification label. To account for the interactional nature of the underlying feature set for classification, participants were labeled as belonging to either a mixed (ASD-TD) or non-autistic control (TD-TD) dyad, resulting in groups of 56 and 32 individuals respectively. Consequently, both interactants within one dyad received the same label, regardless of their individual diagnosis. This labeling procedure was modeled closely to a diagnostic setting in clinical reality, in which only one interactant’s diagnostic status would be at question whereas the other interactant would represent the clinical rater. Linear SVM optimized a linear hyperplane in a high-dimensional data space that maximized separability between individuals belonging to either of the two dyad types (i.e., the support vectors). Based on the trained hyperplane, the data was subsequently projected into the linear kernel space and their geometric distance to the decision boundary was measured, therefore, predicting each participant’s classification. Every participant was assigned a decision score and a predicted classification label.

We built separate models for the synchrony of facial action units (FACEsync; 168 features per individual), head movement (HEADsync; global head movement, as well as pitch, yaw and roll; 56 features per individual), and body movement (BODYsync; 14 features per individual), as well as intrapersonal head-body movement coordination (INTRAsync; 14 features per individual), and individual movement parameters (MovEx; total head and body movement, and facial expressiveness; 6 features per individual). The decision scores of all our base models, as well as the model covering the head region (FACEsync + HEADsync), were subsequently combined in a stacking-based data fusion framework [47] to assess whether a combination of the modalities would result in superior prediction results than the unimodal classifiers themselves.

We additionally conducted supplementary analyses using individual diagnosis as classification label. Results of these analyses can be found in the Supplementary Information S3.6.

### Support vector machine learning analysis

Machine learning analyses were conducted with the toolbox NeuroMiner (Version 1.1; https://github.com/neurominer-git/NeuroMiner_1.1) [48] in MATLAB (Version 2022b) [49]. A repeated, nested, stratified cross-validation (CV) structure was implemented with 11 outer CV folds and ten permutations (CV2) and ten inner CV folds with one permutation (CV1). At the CV2 level, we iteratively held back participants from four dyads as test samples (approx. 9% of data), while the rest of the data (approx. 81%) entered the CV1 cycle, where the data were again split into validation and training sets. Both interactants from a dyad would always remain in the same fold. This nested stratified CV allows for a strict separation between training and testing data, with hyper-parameter tuning happening entirely within the CV1 loop while the CV2 loop exclusively measured the model’s generalizability to unseen data. Additionally, the stratified design ensured that proportion of dyad type in every fold would adequately reflect the proportion of dyad type in the full sample in order to avoid training bias. The five base models were pre-processed and trained separately using LIBLINEAR Support Vector L2-regularized L2-loss classification algorithms (see Supplementary Information S3.1 and S3.2). Given that the current dataset contains a rather high feature-to-sample ratio, this specific algorithm was chosen because of its similarity to LIBSVM but without implementing complex kernels which could potentially result in overfitting. All models were corrected for class imbalance by hyperplane weighting. Balanced Accuracy (BAC = (sensitivity + specificity)/2) was used as the performance criterion for parameter optimization. Statistical significance of the base classifiers was assessed through permutation testing [50]. The permutation testing procedure determines how statistically significant is the model’s performances (i.e., BAC) using the current data compare to models trained on the dataset but with the labels randomly permuted. The permutation test was repeated 1000 times. The significance level was set to *α* = 0.05. In current discussions, an alpha level of .005 has been proposed [51], though the appropriateness of this approach has been called into question [52]. Hence, to reassure statistically rigorous results, we additionally annotated when a significant model’s permutation test result would fail significance using the stricter alpha level. To control for potential bias of the dyadic nature of the data on each model’s significance, each permutation analysis was conducted with both participants of each dyad permuted in pairs according to their dyadic structure. For further details on the permutation testing procedure, see Supplementary Information S3.3. The two stacking models [53] were trained on the resulting decision scores (all base models, facial expression + head motion synchrony) by wrapping them in the identical cross-validation framework as the base models. A L1-loss LIBSVM algorithm with Gaussian kernel was employed to find a parsimonious combination of decision scores which maximized BAC across the C parameter range. For details, see Supplementary Information S3.

## Results

### Base model performances

**Fig. 2:**
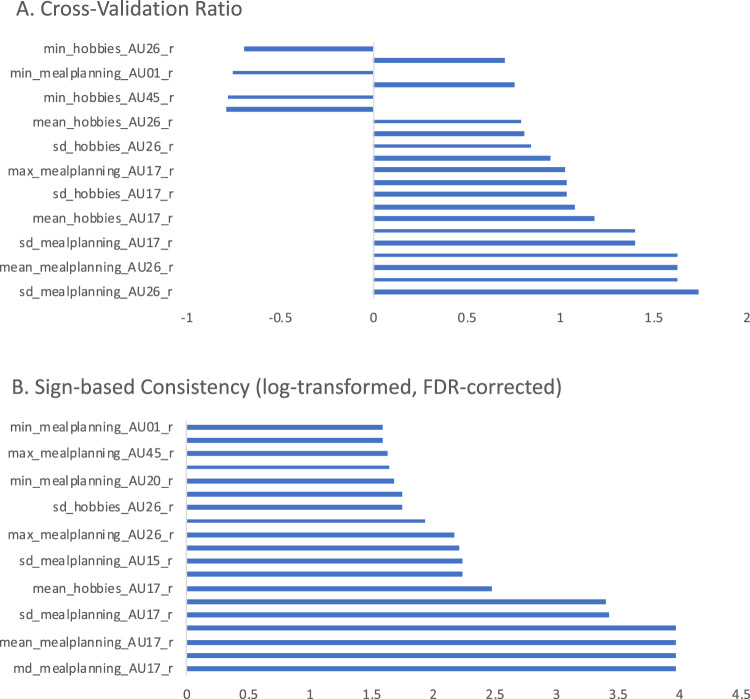
Contribution of features in FACEsync model. Cross-validation ratio of feature weights (**A**) and sign-based consistency (**B**) for the FACEsync model. The features depicted correspond to the person-specific adaptation of intensity of a participant to their dyadic counterpart in the respective facial action units (AU) for either hobbies or mealplanning task (min minimum, sd standard deviation, max maximum).

Using facial action unit (AU) synchrony data, the repeated nested stratified cross-validation FACEsync model yielded a balanced accuracy (BAC) of 79.5%, and an area under the receiver operating curve (AUC) of .82 (*p* < 0.001, also see Supplementary Fig. S9). The contribution of the different features to classification group (Fig. 2) was calculated by feature weights (see Supplementary Information S3.4) and cross-validation ratio. Additionally, the sign-based consistency was explored as an indicator of the feature classification reliability. Assignment to the ASD-TD dyads was mainly driven by features describing an elevated and highly varied extent of adaptation in AU17 (chin raiser) and AU26 (jaw drop). Minimized adaptation in AU01 (inner brow raiser), AU20 (lip stretcher) and AU45 (blink) were indicative of belonging to the TD-TD interaction type. In order to investigate any associations of facial emotion recognition abilities and adaptation behaviors of the different facial AUs, correlation analyses were performed between the decision scores derived from the FACEsync model and accuracy and response time (rt) from the Berlin Emotion Recognition Test (BERT [44]). No significant associations were found (*r*_accuracy_(86) = −0.16, *r*_rt_(86) = 0.13; both *p* = 0.23 after FDR correction).

The model using only head motion coordination data (HEADsync) achieved a BAC of 62.1% and an AUC of 0.64 (*p* = 0.002). Assignment to the TD-TD group was driven by higher values in minimum adaptation of global head movement whereas higher maximum and more variant values for head movement adaptation predicted the ASD-TD group.

The classification model based on upper body movement coordination (BODYsync) predicted dyad origin with a BAC of 56.7% and an AUC of 0.55 (*p* = 0.009). Using a stricter alpha level of 0.005, this model would not be judged as performing significantly better than chance.

Our classification model based on intrapersonal head-body coordination (INTRAsync) performed around chance level with a BAC of 44.2% and an AUC of 0.44 (*p* = 0.994).

The SVM classification model based on features of total head and body movement and general facial expressiveness (MovEx) predicted dyad origin with a BAC of 68.8% and an AUC of 0.75 (*p* < 0.001).

Additional classification metrics for all models can be found in Supplementary Table S6.

### Stacking model

All base model decision scores were extracted and combined into a hierarchical stacking-based fusion framework to assess potential prediction improvements. Combinations of only the head region (FACEsync + HEADsync; BAC = 78.8%, AUC = 0.83), as well as of all modalities (BAC = 77.9%, AUC = 0.85) did not outperform the most predictive base model (FACEsync) with 79.5%.

**Fig. 3:**
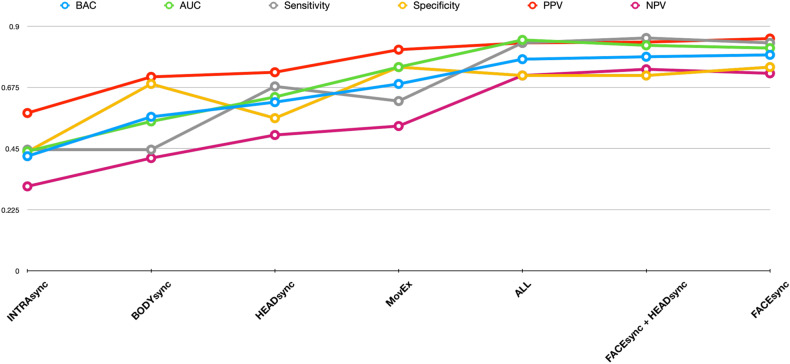
Classification metrics for all base and stacking models. BAC balanced accuracy, AUC area under the curve, PPV positive predictive value, NPV negative predictive value. Models are depicted in the order of lowest to highest performing BAC.

Additional classification metrics of all models are depicted in Fig. 3.

### Classification based on diagnostic group

We repeated all SVM analyses using different labels based on diagnostic groups while ignoring interaction type. These additional analyses were conducted in order to investigate if our collected social interaction data was specific enough to identify an autistic individual, regardless of interaction dyad origin. All models generated inferior prediction accuracies compared to the dyad labeling approach (3.1). Detailed results can be found in Supplementary Information S3.6.

## Discussion

The aim of the current study was to quantify social interaction in ASD for the purpose of automatized diagnostic classification. In this proof-of-concept study, we set out to utilize a dyadic setting for classification of autistic vs. non-autistic interaction based on reciprocity. Participants were filmed conducting two brief conversations about pre-set topics. Using repeated nested cross-validation techniques, we could show that SVM classification models based on different modalities of behavioral reciprocity were sufficient to predict dyad membership to a high degree. Contrary to our hypothesis, combining different non-verbal modalities did not improve overall predictive accuracy. Classification into individual diagnostic groups (ASD vs. TD) based on social interaction data performed worse on all modalities, as well as the model classifying on individual measures of full body movement and general facial expressiveness. This highlights the importance of the social context to capture the manifestation of autistic symptoms.

A model based on reciprocity of facial action units within the interactions showed the best classification accuracy (79.5%) within our sample. When looking more closely at individual feature importance in the facial region, we found heightened and more varied scores for reciprocal adaptation in the AUs chin raiser, jaw drop and lip corner depressor in both tasks to be indicative for classification into the autistic interaction type. This was especially pronounced for the mealplanning task, suggesting higher and more varied synchrony in this task in the ASD-TD interactions. While elevated synchrony in ASD might seem counterintuitive at first glance, especially in light of findings on reduced mimicry in autism [54, 55], taking a closer look at feature importance for the TD-TD group provides a differentiated picture. Participants with higher values for minimum adaptation across all features had an increased likelihood to be classified into the TD-TD group, suggesting a potential floor effect for facial synchrony in this group. Thus, their synchrony did not subceed a certain lower threshold. This was especially pronounced in the action units for inner brow raiser (AU1), lip stretcher (AU20) and blinking (AU45). Additionally, motor synchrony in autistic interactions has previously been found to vary along with the level of autistic traits, social-communicative functioning, and context [19]. The same mechanisms may hold true for mimicry. For example, in a study investigating mimicry in the BERT emotion recognition task, Drimalla and colleagues [56] found significantly more variance in the intensity of facial expressions in autistic participants. Importantly, since machine learning analyses factor in countless interdependencies between features, interpretations based on feature weights should be considered with caution. Nevertheless, the rather high classification accuracy based solely on facial synchrony features found in our study provides valuable implications for future research on classification based on social interactions in an even more ecological setting (e.g., diagnostic assessments via video conferencing).

Interestingly, our model based on measures of individual amount of full body movement and general facial expressiveness (MovEx) was the second-best of the base learners, supporting findings of a characteristic motor signature in autism. For example, Zhao and colleagues [57] investigated head movements in autistic children during live interactions and found aberrances on all three axes. Notably though, our classification model factoring in dyad type, thus, data that included the TD interaction partners, showed superior performance compared to classification based on diagnosis. Hence, interactional aspects also seem to have an association with individual movement features, supporting the hypothesis that intra- and interpersonal adjustment processes are not entirely independent of each other [58].

Contrary to previous findings of high classification accuracy for head and body coordination [24], our model based on this modality performed at a below-chance level, showing low specificity of head-body coordination for autistic vs. non-autistic interaction. However, interpretation should be considered cautiously given the specifications of our experimental setup. Due to our data being collected as part of a larger setup, participants wore wristbands on their non-dominant hand measuring physiological data (see Supplementary Information S1). In order to reduce artefacts in physiological data acquisition, participants were instructed to relax their non-dominant hand in their lap. Arguably, this instruction and setup difference with regards to the previous study could well account for the lack of classification power by intrapersonal coordination in the current study. This is supported by the absence of a significant difference between body synchrony found between our participants’ motion time series and randomly matched time series (see Supplementary Information S2.3).

While our results support previous findings on head motion synchrony as a distinguishing feature of autistic communication [21], combining it with facial expression synchrony did not yield a higher prediction accuracy in a stacking model. This was also the case for our overall stacking model. However, stacking may be able to improve predictive performance of any problem primarily in cases where the underlying data is not well represented by a single model [59], which is not the case in the current study. Furthermore, combining several models with significantly different predictive accuracies might in fact harm overall performance of the stacker. Additionally, if the underlying base models are highly correlated, combining them does not necessarily lead to improved performance [59]. In fact, we did find significant associations of our MovEx model (total head and body movement and general facial expressiveness) with HEADsync for the ASD-TD group (*r* = 0.55, *p* < 0.001), as well with INTRAsync for the TD-TD group (*r* = 0.52, *p* > 0.05; for further details see Supplementary Information S4.5). In our study, we aimed to combine different modalities in a hypothesis-driven way to retain a certain amount of interpretability. We found no added benefit for increasing model complexity. However, it is possible that in order to improve predictive performance of social interactions features, non-verbal aspects of social interaction could be complemented by different modalities in the future, such as speech, eye-movements, physiological or neurological measures. For example, in a recent study conducted by Liao et al. [60], simultaneous measures of EEG, eye tracking and facial expression were assessed of autistic children viewing social and non-social stimuli. The authors found superior prediction accuracies for the combination of behavioral and physiological classifiers.

Notably, there are several limitations within the scope of the present study.

First, the sample size in the current study is limited. To counter this, we implemented a repeated nested cross-validation structure as well as careful feature reduction methods. Nevertheless, our findings should be considered as proof-of-concept and will have to be validated in a larger and external sample, possibly including adults with different psychiatric diagnoses, including comorbidities, to examine specificity within a clinical context more closely and, hence, strengthen the translational aspect [61]. Additionally, regarding the differing incidence rates and possibly phenotypical presentation in males and females with autism, larger samples will allow for thorough analyses of sex and gender effects on social interaction in autism. In any case, we believe that automatic extraction and classification algorithms of social behaviors can support human observation, as they have the possibility to extract behavioral subtleties reliably (e.g., subtle facial expressions [62]), and, thus, could augment diagnostic decision making [63] over and above potential biases. We are convinced that the high scalability of our largely automatized setup can facilitate a simplified data collection process within clinical settings, ideally allowing for cross-site validation approaches which are crucial to the development of reliable clinical prediction models [64]. Second, though interpersonal synchrony has been found to be reduced in interactional dyadic settings independent of partner diagnosis [22], a preference for interactions within purely autistic dyads as compared to mixed interactions has been suggested [12]. This is reflected in theoretical frameworks, such as the “double empathy problem” [65] as well as “dialectic misattunement” [66], specifying autistic impairments to be especially pronounced between people with fundamentally different ways of information processing and interacting. While this underlines the notion of ASD as a social interaction disorder, in a real-world and especially clinical setting this homogenous combination is rarely to be found, which is why this dyad composition was not assessed in this study.

Third, though highly scalable, we relied on different existing computer vision algorithms for our study. On the one hand, this means that the direct comparison of the base models’ accuracies has to be interpreted with caution, as both computer vision algorithms used employ different methods of movement extraction. On the other hand, these algorithms are associated with certain limitations themselves. For example, Motion Energy Analysis (MEA) as a video analysis method has constraints regarding the dimensionality of movement. Because MEA only outputs changes in motion, no specifications regarding direction or magnitude of movement can be made. However, while more distinct investigations of these factors in ASD are certainly desirable, they nevertheless add another layer of complexity to already highly dimensional prediction models. With an increasing feature-to-sample ratio, the ability of ML classifiers to learn more complex relationships may be restricted. Therefore, this was not a focus in our study. Regarding facial expression, a range of AUs and participants had to be excluded due to their extent of missing values within their resulting time series. This was partially due to the participants moving out of the camera frame. Though OpenFace employs person-specific normalization by subtracting a “neutral” face from all other frames of a person, the algorithm is nevertheless reported as potentially less accurate if a face does not show a lot of movement dynamics [67]. Further, within this study the AUs were extracted in a completely automated fashion, without external validation by human coders. While performance accuracy measures for OpenFace are generally favorable compared to other automatic facial expression detection algorithms [45] and this fully automatic approach furthers scalability, nevertheless, it cannot be ruled out that the AUs were not measured accurately, limiting direct interpretations in terms of specific AUs. However, even considering those technical drawbacks, our FACEsync model achieved high classification accuracy. We believe that with the continuing technological developments within computer vision methodology this limitation will likely be overcome in the future.

Lastly, the application of machine learning in clinical psychology and psychiatry is providing novel possibilities for increased precision in individualized diagnosis, prognosis and treatment [68]. However, with increasing model complexity, interpretation of findings and their implications become more challenging. While our findings point to the predictive accuracy of reciprocity in social interactions for autism, future research should aim to gain a greater understanding about the underlying mechanisms of those features. For instance, while we have found high predictive accuracy for an overall estimate of autistic reciprocal interaction within a conversation, a more fine-grained analysis of behavioral synchrony at different time points could shed light on possibly fluctuating interaction dynamics. In addition, while this study mainly explored the use and performance of one of the most widely used machine learning algorithms in psychiatric research [68], there exist a range of other supervised and unsupervised machine learning algorithms that, given a careful cross-validation procedure, tend to perform well with small sample sizes. An additional exploratory analysis using Random Forest Classification is included in the Supplementary Material (Section S4.7). However, to gain deeper understanding of underlying interactions and mechanisms in autistic social interaction, future research should compare the performance indices and feature spaces selected by different algorithms across different samples. Furthermore, interpretable machine learning models could be used in future studies to take feature analysis to the individual level and, thus, study the heterogeneity of ASD in more detail as well as develop more personalized psychosocial interventions.

In this study, we tested adults with autism with a diverse range of cognitive functioning levels, autistic traits and ages. Nevertheless, our SVM models managed to identify participants of an autistic social interaction with high accuracy. While this approach prevents disclosure of the diagnostic status of each individual within a dyad, thus, preserves anonymity, the continuing developments in computer vision prediction models may raise concerns of those affected over the risk of unwilling identification. Hence, it should be emphasized that a professional clinician’s rating is essential for diagnostic decision making in psychiatric care. Consequently, diagnostic prediction models should be viewed as augmenting, rather than replacing diagnostic assessments made by trained clinicians [63]. However, a shift of data collection from traditional questionnaire-based or behavior observation diagnostic tools to objective digital markers will produce sensitive data that needs to be continuously treated with greatest care and data protection standards need to be abided by. Here, automated coding of behaviors is especially beneficial as opposed to manual approaches, allowing for instant anonymization of extracted time series.

Conclusively, using carefully cross-validated ML algorithms, we were able to classify members of autistic and non-autistic dyads based on multiple objective non-verbal measures of reciprocity in naturalistic social interactions. Facial synchrony within the dyad as unit of analysis [29] proved to be the most valuable marker for diagnostic classification of ASD. We are confident that with the growing interconnectedness in psychiatric and computational research, the complexity of social interaction difficulties in autism can be optimally captured.

### Supplementary information

Supplementary Material

## Data Availability

The dataset generated and analyzed during the current study contains clinical information and is therefore not publicly available. They are available from the first author upon reasonable request pending the approval of the coauthors. The preprocessing scripts used during this study are available under https://github.com/jckoe/MLASS-study.
